# Iterative analysis of cerebrovascular reactivity dynamic response by temporal decomposition

**DOI:** 10.1002/brb3.705

**Published:** 2017-07-26

**Authors:** Christiaan Hendrik Bas van Niftrik, Marco Piccirelli, Oliver Bozinov, Athina Pangalu, Joseph A. Fisher, Antonios Valavanis, Andreas R. Luft, Michael Weller, Luca Regli, Jorn Fierstra

**Affiliations:** ^1^ Department of Neurosurgery University Hospital Zurich University of Zurich Zurich Switzerland; ^2^ Clinical Neuroscience Center University Hospital Zurich Zurich Switzerland; ^3^ Department of Neuroradiology University Hospital Zurich University of Zurich Zurich Switzerland; ^4^ Department of Anesthesiology University Health Network University of Toronto Toronto ON Canada; ^5^ Department of Neurology University Hospital Zurich University of Zurich Zurich Switzerland; ^6^ Cereneo Center for Neurology and Rehabilitation Vitznau Switzerland

**Keywords:** blood‐oxygen‐level‐dependent, carbon dioxide, cerebrovascular reactivity, functional magnetic resonance imaging, humans

## Abstract

**Objective:**

To improve quantitative cerebrovascular reactivity (CVR) measurements and CO_2_ arrival times, we present an iterative analysis capable of decomposing different temporal components of the dynamic carbon dioxide‐ Blood Oxygen‐Level Dependent (CO_2_‐BOLD) relationship.

**Experimental Design:**

Decomposition of the dynamic parameters included a redefinition of the voxel‐wise CO_2_ arrival time, and a separation from the vascular response to a stepwise increase in CO_2_ (Delay to signal Plateau – DTP) and a decrease in CO_2_ (Delay to signal Baseline –DTB). Twenty‐five (normal) datasets, obtained from BOLD MRI combined with a standardized pseudo‐square wave CO_2_ change, were co‐registered to generate reference atlases for the aforementioned dynamic processes to score the voxel‐by‐voxel deviation probability from normal range. This analysis is further illustrated in two subjects with unilateral carotid artery occlusion using these reference atlases.

**Principal Observations:**

We have found that our redefined CO_2_ arrival time resulted in the best data fit. Additionally, excluding both dynamic BOLD phases (DTP and DTB) resulted in a static CVR, that is maximal response, defined as CVR calculated only over a normocapnic and hypercapnic calibrated plateau.

**Conclusion:**

Decomposition and novel iterative modeling of different temporal components of the dynamic CO_2_‐BOLD relationship improves quantitative CVR measurements.

## 1 Introduction

Cerebrovascular Reactivity (CVR) describes the degree of Blood Oxygen‐Level Dependent Magnetic Resonance Imaging (BOLD MRI) signal change normalized for the applied vasoactive stimulus, in this study, a standardized iso‐oxic increase in the arterial partial pressure of carbon dioxide (PaCO_2_; Battisti‐Charbonney, Fisher, & Duffin, 2011). Clinically, CVR holds great imaging potential to assess stroke risk (Kuroda et al., 2001; Markus & Cullinane, 2001), chronic structural brain tissue changes (Conklin et al., 2011; Fierstra et al., 2010), and neurovascular uncoupling in brain eloquent areas related to false negative activation on presurgical task related fMRI (Pillai & Mikulis, 2015; Pillai & Zaca, 2011).

Reliably measuring quantitative CVR, however, is challenging due to various dynamic response components contained in the BOLD‐CO_2_ relationship. For instance, delay of the BOLD signal response to PaCO_2_ (i.e., temporal delay)_,_ varies for individual vessel response times and CO_2_ arrival times. Contrarily, static CVR measurements – by using two different steady states, e.g. normocapnia und hypercapnia – only represents the maximal overall response (Vagal, Leach, Fernandez‐Ulloa, & Zuccarello, 2009), thus leaving out important dynamics of the (patho‐) physiological BOLD response.

To better model true CVR response, investigators (Blockley, Driver, Francis, Fisher, & Gowland, 2011; Donahue et al., 2015; Geranmayeh, Wise, Leech, & Murphy, 2015; Halani, Kwinta, Golestani, Khatamian, & Chen, 2015; Poublanc et al., 2013) have aimed to define the arterial arrival time of PaCO_2_ by applying maximum Pearson product‐moment correlation coefficient between CO_2_ and BOLD signal response, assuming a constant rate of vascular dynamic response over the brain. In this instance, the delay inducing the maximum correlation is considered the arterial arrival time. The assumption of constant rate of vascular dynamic response over the brain has recently been challenged. Already for healthy vessels there is a different vascular response to CO_2_ between subjects (Regan, Duffin, & Fisher, 2013; Regan, Fisher, & Duffin, 2014) as well as for white and gray matter vessels within a single subject (see Figure 4 from Bhogal et al., 2015). Besides, even at a similar rate of dynamic vascular response, the maximum correlation does not only incorporate arterial arrival time of CO_2_, it also integrates part of the vascular dynamic response function to PaCO_2_ (Bhogal et al., 2015). Therefore, calculating CVR after maximum correlation may result in suboptimal separation of CO_2_ arrival time and consequently a location‐dependent erroneous CVR interpretation.

Using an iterative analysis, we model and separate different components of the dynamic CO_2_‐BOLD relationship to acquire more detailed information about the dynamic vessel response times to CO_2_ between different brain regions on a voxel‐by‐voxel basis. By redefining the CO_2_ arterial arrival time, we are able to provide a more optimal dynamic CVR calculation for (patho‐) physiological delayed arrival time for every voxel in the brain. Additionally, this method allows for BOLD MRI‐based static CVR measurements.

## 2 Methods

### 2.1 Data selection and acquisition

This study was approved by the cantonal ethics board of the Canton of Zurich (KEK‐ZH‐Nr. 2012‐0427). Normal datasets from 25 subjects were selected from an ongoing prospective study on BOLD‐MRI CVR at our institution. Exclusion criteria were the presence of severe cardiopulmonary disease, MRI contraindications, age <18 old or the inability or refusal to sign informed consent. For this analysis, two subjects with unilateral carotid artery occlusion (CAO) with subsequent unilateral hemodynamic impairment, as diagnosed by H_2_O Positron Emission Tomography (PET), were selected from the database based on the following inclusion criteria: presence of unilateral ICA occlusion, normal anatomical scans without other neurological abnormalities. Inclusion criteria for the 25 reference subjects were: No medication use; no medical history of neurological disease and neurological symptoms at day of scanning.

Data was acquired on a 3 Tesla Skyra VD13 (Siemens, Erlangen, Germany) with a 32‐channel head coil. Whole‐brain BOLD volumes were collected with the following parameters: an axial 2D EPI BOLD fMRI sequence planned on the ACPC line plus 20° on a sagittal image with voxel size: 3 × 3 × 3 mm^3^, acquisition of matrix 64 × 64 × 35 slices with ascending interleaved acquisition, slice gap 0.3 mm, GRAPPA factor 2 with 32 ref. lines, Repetition Time (TR)/ echo time (TE) 2000/30 ms, flip angle 85°, bandwidth 2368 Hz/Px, Field of View 192 × 192 mm. 200 volumes were acquired.

A high‐resolution 3D T1‐weighted MPRAge image was also acquired with the same orientation as the fMRI scans for overlay purposes. The acquisition parameters were: voxel size 0.8 × 0.8 × 1.0 mm with a Field of View 230 × 230 mm and Resolution of 288 × 288. 176 slices per slab with a thickness of 1 mm, TR/TE 2200/5.14 ms, TI 900 ms, flip angle 8°.

### 2.2 Iso‐oxic CO_2_ stimulus

**Figure 1:**
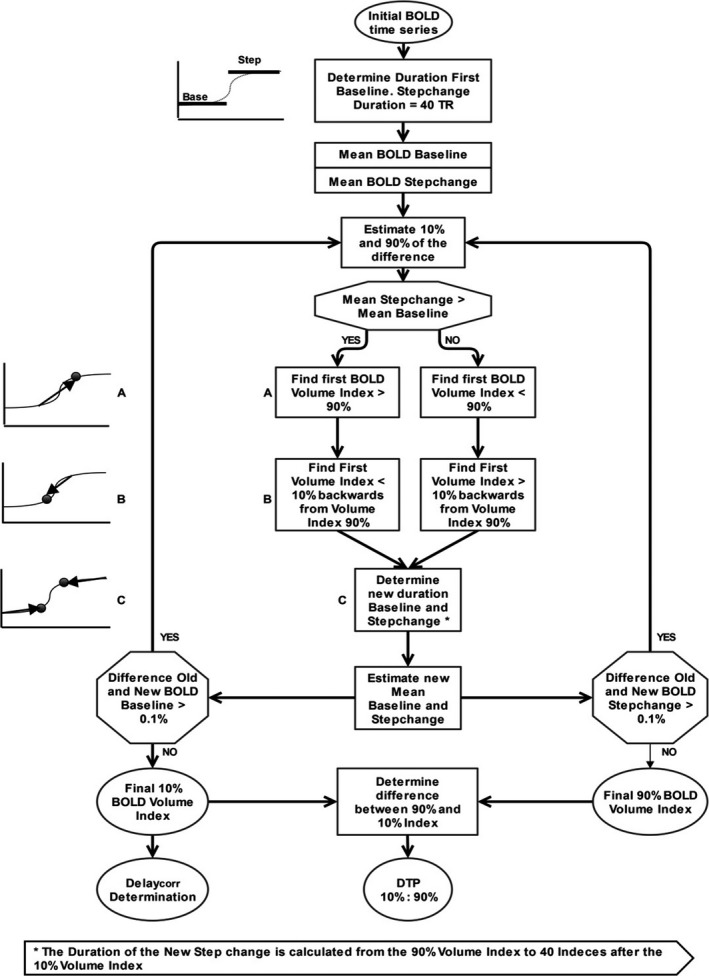
Flow Chart DTP calculations. Flow charts of the iterative DTP determination on a voxel‐wise basis. The temporal decomposition uses a maximum of 15 iterations. DTP, Delay to Plateau

End‐tidal partial pressure of oxygen (PetO_2_) and carbon dioxide (PetCO_2_) were precisely controlled and targeted using a preprogrammed sequence, executed by a custom built computer controlled gas blender (Prisman et al., 2008; RespirAct^TM^, Thornhill Research Institute, Toronto, ON, Canada) with prospective gas targeting algorithms described by Slessarev et al. (2007). Specific details and novelties of this technique as compared to other vasoactive stimuli have been described more specifically in a previous publication (Fierstra et al., 2013). The preprogrammed sequence consisted of an initial 100 s baseline P_et_CO_2_ at 40 mmHg, after which a P_et_CO_2_ step was increased to 10 mmHg above baseline for 80 s and a return to baseline for 100 s, before free breathing was restored. Iso‐oxia was maintained at 100 mmHg (Figure 1 – green line).

### 2.3 Data preprocessing

#### 2.3.1 Spatial preprocessing

Anatomical and functional images were preprocessed using Statistical Parametric Mapping 12 (SPM 12, Wellcome Trust Centre for Neuroimaging, Institute of Neurology, University College London, UK). First BOLD images were realigned to the mean BOLD image. The high‐resolution MPRAge T1‐weighted image was coregistered to the mean BOLD image and probability maps for gray matter, white matter, cerebrospinal fluid, skull, skin and air were generated. Left and right hemispheres were manually segmented. For construction of normalized volumes, functional and anatomical maps were normalized to the MNI template using a non‐linear transformation into MNI space. After removal of all voxels, excluding the white and gray matter voxels to decrease the partial volume effects of neighboring CSF voxels, the functional images were spatially smoothed with a Gaussian kernel of 8 mm full width at half maximum.

#### 2.3.2 Temporal preprocessing

**Figure 2:**
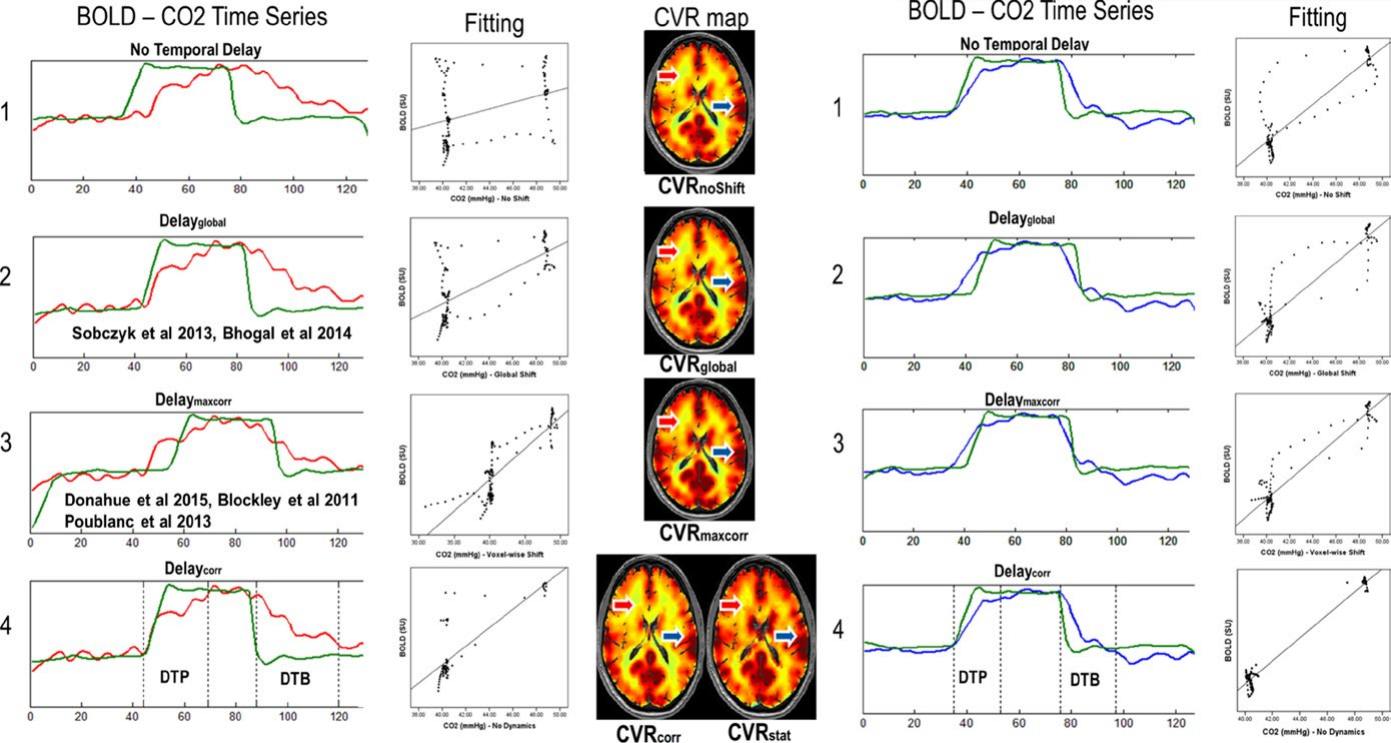
CO_2_ time evolution vs. BOLD signal in two illustrative voxels. For each processing method, the shifted CO_2_ timeline (green) is plotted against the BOLD signal time series of two random voxels (one in the white matter: red, and one in the gray matter: blue) together with the linear fitting of the BOLD vs. CO2 scatter plot and the respective CVR map. In figure 2.1, the CO_2_ time series correspond to CO_2_ time course without time shift. figure 2.2 shows a single global CO_2_ time shift for the whole‐brain (Delay_global_) and figure 2.3 correlation CO_2_ time shift (CO_2_‐BOLD) on a voxel‐wise basis after maximum correlation (Delay_maxcorr_); figure 2.4 shows the respective duration of the DTP and the DTB for both voxels with the dynamic (CVR_corr_) and static (CVR_stat_) CVR maps. The fitting of figure 2.4 is based on CVR_stat_, scatter plot which shows clearly removal of the transition phases between the two static states. The shifts of the CO_2_ timeline are: Delay_global_: red: 7 TRs, blue: 7 TRs; Delay_maxcorr_: red: 20 TRs, blue: 5 TR; Delay_corr_: red 9 TR, blue: 1 TR. CO_2_, Carbon dioxide; BOLD, Blood‐oxygen‐level‐dependent; CVR, cerebrovascular reactivity; DTP, Delay to Plateau; DTB, Delay to Baseline

The MR data sets were further preprocessed with custom MATLAB R2013b routines (The MathWorks, Inc., Natick, Massachusetts, United States; http://www.mathworks.com/). A low band‐pass filter with a filter cut‐off frequency of 0.125 Hz was applied to the MR data following Duffin et al. (2015) (Figure 2). Thereafter, the BOLD time series were temporally smoothed by a 16 dynamics (6%) local regression using weighted linear least squares and a second‐order polynomial model with assignment of lower weight to outliers (robust Loess method). This procedure did not induce any time shift in the data. Last, we linearly detrended the BOLD time series. Additionally, the PetCO_2_ trace was resampled to match the TR of the BOLD data.

### 2.4 CO_2_ arrival time (Temporal Delay)

We used three different types of CO_2_ arrival times (Temporal Delay) to match the CO_2_ to the BOLD time course:

1. a global CO_2_ shift for the whole‐brain (Delay_global_), the most commonly used method (Bhogal et al., 2015; Sobczyk, Battisti‐Charbonney, Fierstra, et al., 2014; Sobczyk, Battisti‐Charbonney, Poublanc, et al., 2014).
2. a voxel‐wise CO_2_ shift (Delay_maxcorr_) which has been proposed by Poublanc et al., 2013 (Blockley et al., 2011; Poublanc et al., 2013) and
3. our novel method for voxel‐wise CO_2_ shift corrected for dynamical processes (Delay_corr_).

For the first two types (Delay_global_ and Delay_maxcorr_), we implemented a linear least‐square best fit of these CO_2_ time course to the BOLD signal for maximum cross‐correlation (Pearson Correlation). The lower and upper ranges for the delay determination were −10 and 50 TR (80 s). We did allow for a small negative cross correlation. An upper limit of 50TR was chosen since a delay exceeding 50 TR(100 s) was considered physiologically improbable. The delay with the maximum Pearson correlation was used to calculate the CVR maps, see section 2.8.

### 2.5 Delay to plateau

To characterize the dynamic response we used the ten‐to‐ninety (10% and 90%) rise time duration. Conceptually such a method is used to determine the transition duration between two steady states for instance in electrophysiology (Floyd, 2014). We applied a similar concept to determine Delay to Plateau (DTP), defined as the duration between the 10% (i.e., start) and 90% (i.e., stop) of the dynamic BOLD signal response to a rapid (pseudo square wave) P_et_CO_2_ increase. The substantial difference with standard rise time calculations is that the BOLD signal either increases or decreases to a P_et_CO_2_ increase. The calculations, done on a voxel‐wise basis, are explained in the flow chart in Figure 1. The maximal number of iterations was 15.

### 2.6 Corrected delay

Corrected Delay (Delay_corr_) values were calculated as followed:

$${\text{Delay}}_{\mathrm{corr}}=\left({\text{Volume}}_{10\%}-{\text{Volume}}_{\mathrm{start}}\right)*\text{TR}$$

where Volume_10%_ is the index of the BOLD volume where the BOLD signal increases by 10% – at the beginning of the DTP. Volume_start_ is the BOLD volume index with the computer driven start of the CO_2_ step change minus 10 TR. In determining Volume_start_, we assumed a negative of 10 TR to correct for any delay between the display of CO_2_ on the computer and the actual CO_2_ inspiration, For each patient, we determined the fastest responding voxel and corrected the other voxels accordingly. This allows for optimal inter‐subject analysis. Consequently, Delay_corr_ is a voxel‐wise corrected temporal delay in seconds between these two BOLD volumes indexes, corrected with the delay of the fasted responding voxel.

### 2.7 Delay to baseline

Delay to Baseline (DTB) is defined as the time between the 90% to 10% signal return to baseline after the CO_2_ step change. DTB is comparable to the commonly used “decay time”. However, while decay time is usually seen as the signal drop between fixed percentages, DTB includes calculations for either increasing or decreasing signal responses, that is return to baseline.

For calculating DTB, the dynamic response from the end of the step change in CO_2_, a similar approach was used as for the DTP. We first determined the 10% Volume Index in a similar fashion as we did the 90% Volume Index for DTP. Thereafter, we determined the first Volume Index with BOLD signal intensity >90%. Similar to DTP, this process was also reiterated. The amount of indexes between the 90% and 10% Volume Indices, multiplied by two, is determined to be DTB.

### 2.8 CVR calculations

After shifting the P_et_CO_2_ time course, P_et_CO_2_ was regressed against the BOLD signal time course using a linear least square fitting to calculate CVR, defined as:

$$\text{CVR}=\frac{\%\Delta \text{BOLD}}{\Delta {\text{CO}}_{2}\text{in mmHG}}$$

where %ΔBOLD is the percent of BOLD signal change and ΔCO2 in mmHg is the CO2difference. For comparison, CVR calculations were done with BOLD time series versus the global shifted CO_2_ time series and a shifted CO_2_ time series on a voxel‐per‐voxel basis (Delay_maxcorr_) to create CVR_global_ and CVR_maxcorr_ (See Figure 2; Poublanc et al., 2013; Geranmayeh et al., 2015).

Both CVRs and Delays were color coded and presented as an overlay on the high resolution T1‐weighted image, only to include voxels passing the 0.9 probability threshold in the combined gray and white matter probability map.

### 2.9 Corrected CVR and static CVR

Corrected CVR (CVR_corr_) is calculated in a similar fashion as CVR_maxcorr_ with the difference that Delay_corr_ is used as the temporal voxel‐wise CO_2_ shift.

Static CVR (CVR_stat_) differs from CVR_corr_ as the DTP and DTB periods were excluded from the calculations to determine maximal CVR response by only using plateau phases (here isocapnic and hypercapnic plateau phases; See Figure 2).

### 2.10 Reference atlases and Z maps

Sobczyk, Battisti‐Charbonney, Fierstra, et al. (2014), Sobczyk, Battisti‐Charbonney, Poublanc, et al. (2014) and Poublanc, Crawley, Sobczyk, Montandon, et al. (2015), have previously defined the approach to generate CVR reference atlases, with increased sensitivity for impaired CVR. We have created our reference atlas in a similar fashion. For CVR_corr_, CVR_stat_, Delay_maxcorr_, Delay_corr_, DTP and DTB, the mean and standard deviation over the 25 datasets were calculated to establish the reference atlases.

After creation of each reference atlas with standard deviation, we calculated the Z scores on a voxel‐wise basis as followed (Sobczyk, Battisti‐Charbonney, Fierstra, et al., 2014, Sobczyk, Battisti‐Charbonney, Poublanc, et al., 2014):

$$z-\text{score}=\frac{X-\mu }{\sigma }$$

where value Z is the score for the aforementioned variable (e.g., CVR_corr_ CVR_stat_, etc.), X is the voxel value for the given variable, μ is the mean value for the given variable of our reference atlas for that voxel and σ is the standard deviation for the given variable in that voxel. Abnormal voxels were defined as: | z scores | > 2σ, which implies a t‐test value smaller than 0.05.

Z‐maps were color‐coded and include just voxels with values outside the normal variation range.

## 3 Results

### 3.1 Decomposition of dynamic temporal components

For our iterative analysis we separated the dynamic temporal components and named them as follows: CO_2_ arrival time *– temporal delay‐*, Delay to Plateau, Delay to Baseline.

Figure 2 illustrates the concepts of different CO_2_ arrival times and DTP and DTB for a gray matter and a white matter voxel of an exemplary subject.

As becomes apparent, Delay_global_ is too short for deep white matter voxels (red – Figure 2.2), and too long for gray matter and subcortical voxels (blue – Figure 2.2). Delay_global_ calculations often match a gray matter maximum correlation more optimally. The implementation of a voxel‐wise delay results in improved CVR contrast mainly in the white matter.CO_2_ time series shifted with Delay_maxcorr_ in Figure 2.3 partly corrects the aforementioned limitations inherent to Delay_global_. As Delay_global_ calculations often match a gray matter maximum correlation, the implementation of a Delay_maxcorr_ results in heightened CVR contrast mainly in the white matter.Beside the CO_2_ arrival time, Delay_maxcorr_ also fits the two dynamic vascular responses (DTP and DTB), which causes Delay_maxcorr_ to be a mix of multiple dynamic parameters. With an optimal fit for every voxel, this falsifies CVR calculation since the BOLD signal time response precedes the CO_2_ increase, which is physiologically impossible. Figure 2.4 shows the BOLD signal time series versus CO_2_ time series shifted with the novel Delay_corr_. The dotted lines represent the 10% and 90% ranges of the BOLD signal of DTP and DTB for these voxels. Additionally, a dynamic (CVR_corr_) and static (CVR_stat_) CVR map are shown. The CO_2_ increase corresponds precisely to the beginning of the BOLD signal change whereas the end of the pseudo square wave of CO_2_ corresponds to the beginning of the BOLD signal decrease.

**Figure 3:**
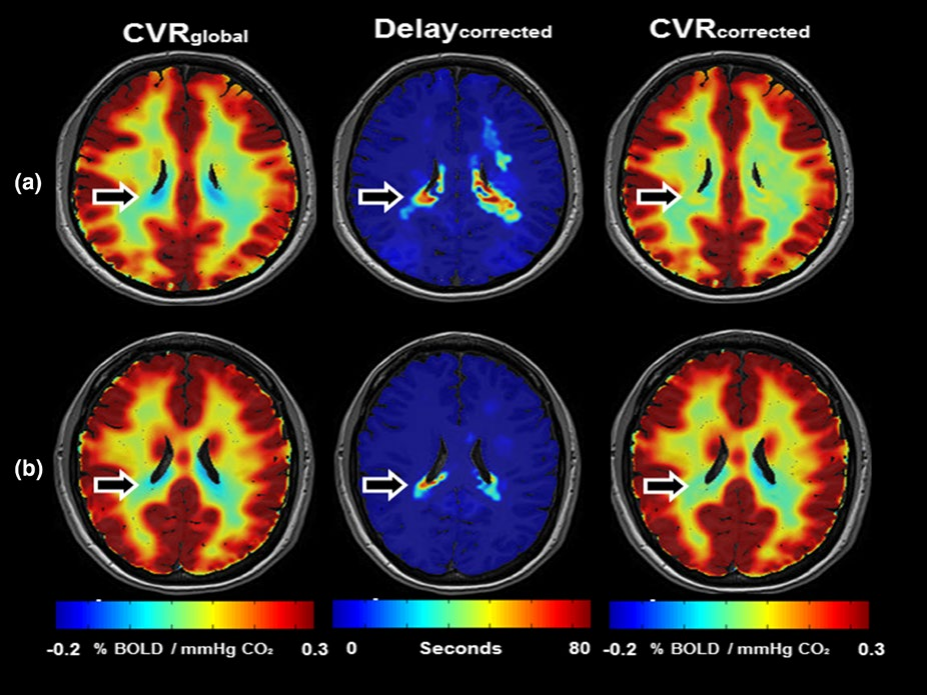
Effect of arterial arrival time on CVR calculations. (a–b) shows a single axial slice of 2 CVR maps (CVR_global_ and CVR_corr_) and a Delay_corr_ map of two representative subjects (age 24 and 26 respectively). CVR is color‐coded between −0.2% and 0.3% to increase CVR contrast and highlight changes. The Delay_corr_ is presented as an overlay on a T1‐weighted image and color‐coded between 0–80 s. Although subtle, it is obvious that in areas with prolonged Delay_corr_ negative CVR findings with CVR_global_ are corrected after CVR_corr_ calculation. Moreover, noticeable is that CVR contrast does not increase in all regions of the brain after Delay_corr_ implementation. In areas in the white matter without prolonged CO_2_ arrival time, the Delay_global_ presents a Delay value more closely matching the Delay_maxcorr_ then the Delay_corr_. This results in an increased correlation between BOLD and CO_2_ time series, enhancing CVR contrast. However, as is shown in Figure 2, this CO_2_ arrival time also incorporates part of the dynamic response, which results in erroneous CVR calculations.

Removing DTP and DTB from the dynamical considerably improves the fit which we termed CVR_stat_. Illustrative functional maps of two subjects can be found in Figure 3a,b. Furthermore we have included illustrative axial slices of DTP, DTB, Delay_maxcorr_, Delay_corr_, CVR_corr_ and CVR_stat_ for both subjects in the supporting information.

### 3.2 Reference atlases

For the formation of the reference atlases for DTP, DTB, Delay_maxcorr_, Delay_corr_, CVR_corr_ and CVR_stat_, a total number of 25 subjects (age 34.2 ± 11.8) were included. Subject characteristics and extended slices of all six reference atlases are given in the supporting information (see Table S1 and Figure S1).

The average whole‐brain DTP and DTB of our reference cohort are 25.1 ± 3.9 s, respectively 29.3 ± 4.5 s.

The overall Delay_maxcorr_ over the whole‐brain is 20.1 ± 13.8 s with a large difference between gray and white matter (15.9 ± 12.3 respectively 25.5 ± 17.2 s). The white matter shows higher values for the corpus callosum, the deep white matter, and the periventricular region. Increased Delay_maxcorr_ is also found around the fronto‐basal and medio‐temporal regions. These delay values are partly modified when investigating the Delay_corr_. Delay_corr_ in most regions varies between 1 and 3 s with an average of 2.4 ± 2.4 Only a slight increase in the deep white matter and periventricular area can be observed (Figure S2). This renders Delay_corr_, in combination with a small standard deviation very sensitive to detect abnormal voxels. Overall CVR values for CVR_corr_ are generally smaller than CVR_stat_ (0.26 ± 0.11 vs. 0.33 ± 0.14).

From the reference atlas, it becomes apparent that the DTP and DTB differences between individual subjects are fairly small (on average 25.3 ± 4.0 vs. 28.8 ± 4.5 s).

### 3.3 Functional maps of patient datasets

**Figure 4:**
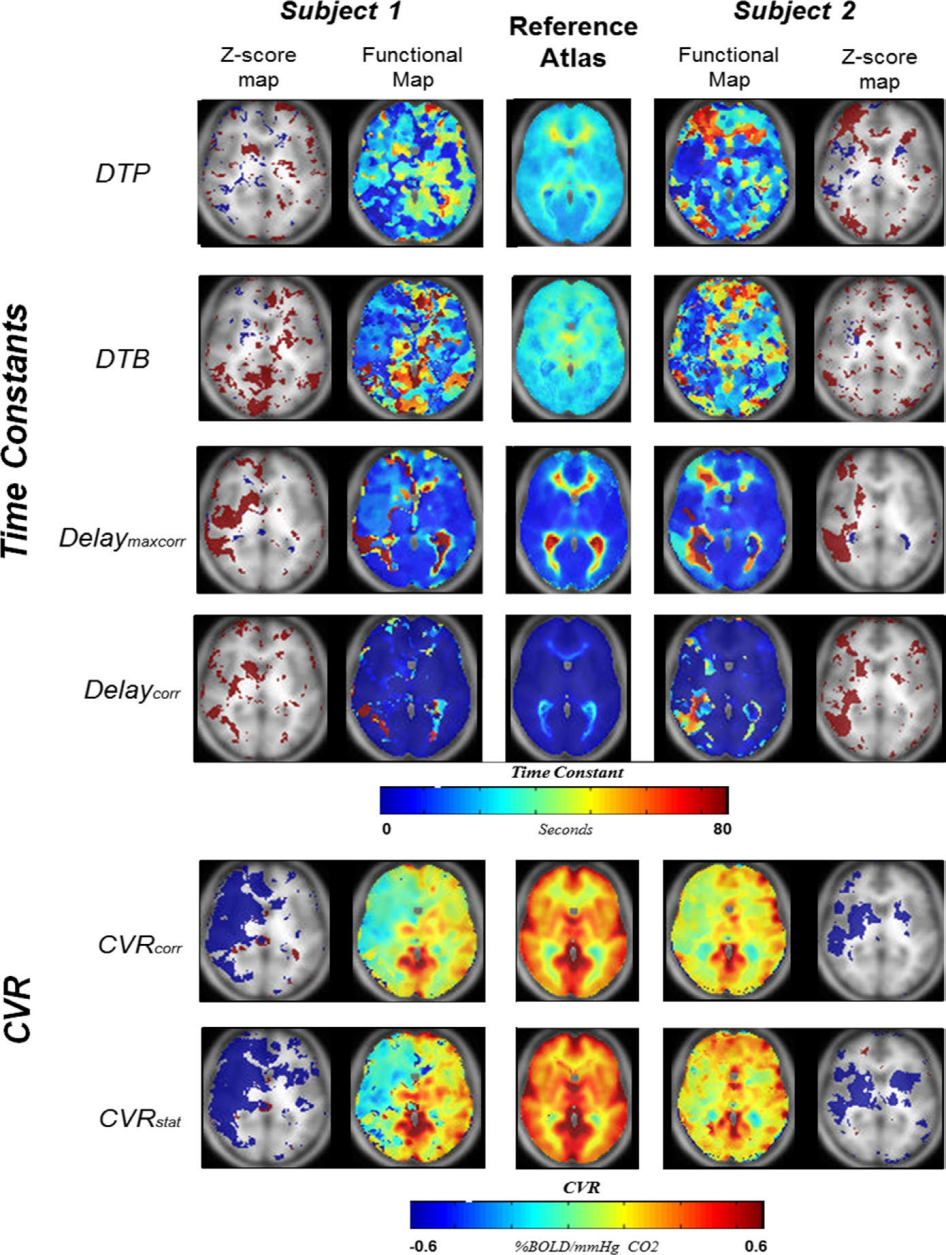
Two illustrative patients. Axial slices of 4 time constant maps and two CVR maps of two patients with unilateral internal carotid artery occlusion. The center images reflect the reference atlases for each parameter. The four time constant maps include DTP, DTB, Delay_maxcorr,_ and Delay_corr._ The time constant maps are color‐coded between 0 and 80 s. The CVR maps shown are the CVR_corr_ and the CVR_stat and_ are color‐coded between −0.6% and +0.6% BOLD signal change per mmHg CO_2_. The two outer images are z‐score maps for abnormality assessment. Only voxels surpassing −2σ (Blue) or +2σ(Red) are shown in this image. CVR, cerebrovascular reactivity; DTP, delay to plateau; DTB, Delay to Baseline

For two subjects with unilateral ICAO an exemplary BOLD‐derived functional axial slice of each parameter in combination with an axial slice of the reference atlas and abnormality analysis is given in Figure 4. The outer left and right images present z‐score maps to visualize voxel‐by‐voxel abnormality deviation from normal. In accordance with Sobczyk, Battisti‐Charbonney, Fierstra, et al. (2014), Sobczyk, Battisti‐Charbonney, Poublanc, et al. (2014) we determined the voxel to be abnormal with | z score | > 2σ. For clarification only abnormal voxels are presented with a color‐coded overlay (−2σ = blue, +2σ = red).

Delay to Plateau and DTB in these subjects show a sensitive modified pattern relative to the reference atlas. Subject 1 shows a large area of paradoxical (negative) CVR in the medial cerebral artery (MCA) and anterior cerebral artery (ACA) areas of the right hemisphere, which are depicted in blue. In the CVR impaired areas, the DTP appears to be prolonged in the area with paradoxical CVR, as well as in large areas of the contralateral hemisphere. Interestingly, the DTB is seemingly shorter in the paradoxical CVR impaired area and longer in surrounding tissue. Together a prolonged DTP and shortened DTB result in a normal Delay_maxcorr_.

In subject 2, in the right sided areas exhibiting impaired CVR, Delay_maxcorr_ is highly influenced by the dynamics of DTP and DTB, while posteriorly to the impaired CVR an increased Delay_corr_ is also present.

Both subjects experience abnormal voxel increases in their time constant parameters at the borders for the impaired CVR area. Subject 2 also shows abnormal decrease within the affected area.

For subject 2, after removing the dynamic components, CVR_stat_ shows a stronger increase in the ACA territory on the right. This is strongly related to the increased DTP found in this patient. For the z‐score map, however, in the ACA only a small correction is observed, whereas the abnormal region in the MCA territory is enlarged.

Both patients show extensive and numerous abnormal z‐CVR‐score regions in the contralateral “unaffected” hemisphere illustrating that a unilateral vasculopathy may affect the entire brain.

## 4 Discussion

We present a novel CVR modeling by decomposing iteratively different dynamic components of the CO_2_‐BOLD relationship thereby acquiring specific information about the varying vessel response times and CO_2_ arrival times – that is temporal delay – on a voxel‐by‐voxel basis. We have characterized a new optimal variant of the CO_2_ arterial arrival time (Delay_corr_), and introduced novel functional parameters, DTP and DTB. This method by itself further improves quantitative CVR measurements, and patient specific voxel‐wise parameter probability can be expressed in statistical z scores by matching it to a reference atlas.

### 4.1 Delay to plateau and delay to baseline

DTP characterizes the duration of the BOLD signal evolution in response to a pseudo square wave of CO_2_ (rapid stepwise CO_2_ increase of 10 mmHg), while DTB represents the dynamic return duration to the calibrated baseline from the end of the CO_2_ pseudo square wave (rapid stepwise CO_2_ decrease of 10 mmHg). DTP and DTB are modifications of the widely adopted – “rise time” and “decay time” neuroimaging parameters.

Our findings of different dynamic vascular responses in gray matter and white matter are in good agreement with findings of other groups using CO_2_ as the vasoactive stimulus (Andrade et al., 2006; Bhogal et al., 2015; Rostrup et al., 2000). The subtle duration differences, seen between the subjects presented in Figure 2, have also been described by Transcranial Doppler studies investigating the dynamic response of healthy subjects to CO_2_ in the MCA territory (Regan et al., 2013, 2014). These studies also found a fast initial response and a delayed secondary response as we observed in both voxels in Figure 2. We also found similarities between our DTP and BOLD‐time‐to‐peak measurements done by Donahue et al. (2013) in healthy subjects.

Recently, Duffin et al. (2015) and Poublanc, Crawley, et al. (2015), determined the dynamic vascular response velocity by convolving a controlled PetCO_2_ stimulus trace, with either the Hemodynamic Response Function (HRF) or a mono‐exponential dispersion function. Their dynamic vascular response velocity maps (termed Tau and Phase) are fairly similar to our DTP map with clear distinctions between short vascular responses in the gray matter and increasingly prolonged dynamic vascular responses from subcortical to deep white matter. The limitation of combining both dynamic patterns in one parameter (i.e., Tau and Phase) automatically results in an abnormally defined voxel, even if only one parameter is prolonged or shortened; This limitation can be observed in Figure 4 – Subject 1 as well as in our reference atlas.

Another caveat of previous studies is the inaccurate correction for arterial arrival time of CO_2_ by Delay_global_. The average BOLD signal contains both gray as well as white matter, which encompasses the majority of brain volume and therefore arterial arrival times and dynamic vascular responses of white matter voxels are strongly represented in the average BOLD signal. As BOLD signal amplitude change in the white matter is known to be lower, the result is an overestimation of gray matter CO_2_ arrival time and underestimation of the arrival time in parts of the white matter, as becomes apparent in Figures 2 and 3. Consequently, convolving CO_2_ with an incorrect arrival time will result in erroneous dynamic vascular response calculations. With our novel method the different vascular response times to PaCO_2_ in distinct parts of the brain, can be quantified voxel‐by‐voxel.

### 4.2 CO_2_ arrival time

Earlier studies determined the arterial arrival time of CO_2_ or Delay_maxcorr_ with maximum correlation calculations and showed an improved explained variance (Andrade et al., 2006; Donahue et al., 2015; Geranmayeh et al., 2015). This erroneously created the issue of BOLD signal increase prior to CO_2_ arrival, which is highly improbable physiologically (see Figure 2.3).

Based on the start of the dynamic vascular response we discarded the concept of Delay_maxcorr_ and created the term Delay_corr_. Delay_corr_ is an improved surrogate of cerebral arterial arrival time of CO_2_. Figure 2 clearly presents Delay_corr_ as the better arterial arrival time definition. The optimization of CO_2_ arrival time by Delay_corr_ can be observed as the parallel increase of BOLD signal and CO_2_ time series, as well as the parallel decrease in BOLD and CO_2_ after the step‐function CO_2_ decrease. This confirms Delay_corr_ primarily reflecting the arterial arrival time without the vascular dynamic response. Large differences in Delay_corr_ can be seen between gray matter and subcortical white matter versus the deep white matter. Gray matter and subcortical white matter are quickly perfused by larger arteries with sufficient anastomosis while in deep white matter the vasculature consists of smaller end‐arteries (Bhogal et al., 2014). For healthy subjects, the use of Delay_global_ results in erroneous findings of steal phenomenon that are only partially corrected if the correct arrival time is applied (Figure 3). Thomas et al. (2013) showed that an adequate CO_2_ stimulus will result in negative CVR due to high signal CSF displacement by vasodilatory response. This could explain negative CVR periventricular after correction with Delay_corr_.

Furthermore, Delay_corr_ also shows a better correspondence to bolus arrival time (BAT) in Dynamic Susceptibility (DSC) MRI or Arterial Spin Labeling (ASL) (Donahue et al., 2014; MacIntosh et al., 2010; Poublanc et al., 2013). Poublanc et al. found a tenfold difference in BOLD Delay_maxcorr_ and DSC MRI derived BAT with BAT values between 0 and 1 s. Moreover Donahue et al. (2014) found BAT times determined with ASL comparable to DSC BAT times found by Poublanc et al. As compared to our Delay_corr_ data, DSC BAT and ASL BAT are marginally shorter. Indeed, the reference/start point, with ASL, is the time and location of water labeling, which can be used as a reference for signal changes measured in different regions in the brain. With our method, the starting point is the PetCO_2_ trace– thus there is a delay from the time the gas is inhaled to the time that the trace is measured by the system with the BOLD signal potentially reacting in between. By using a small negative delay and adjusting the delay to the fasted responding voxel, we corrected for all these factors.

This minor prolongation of Delay_corr_ relative to DSC/ASL BAT is most likely related to a limitation in the calculations due to TR shift and the TR relative discretization noise.

### 4.3 Dynamic versus static CVR calculations

Additionally, we calculate CVR_stat_ based on the calibrated steady‐state CO_2_ and BOLD components of the dynamic CO_2_‐BOLD relationship as a static CVR. Maps created with CVR_stat_ exhibited improved contrast over the whole brain. These maps are based on a vascular steady state, as was also demonstrated by Regan et al. (2014) using transcranial Doppler and isoxic static CO_2_ CVR calculations. It is clear that dynamic CVR calculations are based upon multiple different parameters, all affecting CVR in their own way. Therefore, CVR_stat_ may be more straightforward when comparing differences between subjects because it eliminates the need for a linear evolution during the DTP and DTB times of the BOLD signal response to a hypercapnic challenge (Battisti‐Charbonney et al., 2011; Bhogal et al., 2014). Still, CVR_stat_ does not remove the need to remain within the CO_2_ range, where the BOLD signal is linearly dependent upon PetCO_2_.

Static CVR calculations with BOLD has been previously introduced by Poublanc, Crawley, et al. (2015). Their steady state CVR is determined by convolving CO_2_ with a parametrized hemodynamic response function to best fit the BOLD time series on a voxel‐wise base. This will result in CO_2_ time series, very similar to the BOLD time series, making all calculations more or less linear and therefore static. However, problems may arise when interpreting this steady state CVR. A controlled stimulus is used because of its potential to precisely alter PetCO_2_ by prospecting end tidal targeting with high correspondence to PaCO_2_ (Prisman et al., 2008). Therefore, modeling PetCO_2_ values during the dynamic range to best fit the BOLD time series will result in a fictitious CVR, not corroborated by true underlying PaCO_2_ values.

Another technique for static or time independent CVR calculations is implementation of a controlled ramp protocol (Mutch et al., 2012; Sobczyk, Battisti‐Charbonney, Fierstra, et al., 2014; Sobczyk, Battisti‐Charbonney, Poublanc et al., 2014). Under controlled CO_2_ changes it can be argued that a ramp protocol with a slowly progressive CO_2_ increase can induce vascular dilatation throughout the brain with enough delay to level out DTP. Therefore, a ramp protocol can result in a better time independent – static – CVR calculation without loss of power. However, this is only justified if the ramp protocol is within linear range of the BOLD‐CO_2_ sigmoidal curve (Sobczyk, Battisti‐Charbonney, Fierstra, et al., 2014; Sobczyk, Battisti‐Charbonney, Poublanc et al., 2014). Outside this linear range, the BOLD versus CO_2_ evolution needs to be considered to calculate CVR meaningfully.

### 4.4 Limitations

The correct DTP and DTB determination needs the use of a stepwise CO_2_ change. A slower CO_2_ change would increase DTP and DTB and not allow a correct impulse response function/rate determination. In case of non‐stepwise PetCO_2_ trace, no correction can be made using, for example, the rise and decay constants of the PetCO_2_, as the higher frequencies would not be present in the stimulus.

For these calculations PaCO_2_ needs to be stable. Suboptimal control of the physiological parameters will result in either upward or downward CO_2_ drifts. Consequently, these calculations cannot be done using a non‐standardized CO_2_ stimulus.

An overall limitation for CO_2_ arrival time calculations with fMRI is the temporal discretization on the TR. Our TR of two seconds could be shortened using Multiband acquisition (Setsompop et al., 2012).

Last, the younger cohort included in the reference atlas could have confounded our z‐score calculations, as age related CVR changes have been described (De Vis et al. 2015, Bhogal et al. 2016).

### 4.5 Caveats of the current study

We do not determine or calculate the underlying changes in blood flow, nor measure cerebral flow directly. The determined components (i.e., DTP and DTB) are transient response durations that includes all factors of the dynamic vessel response, including change in cerebral blood flow. We characterize the vascular BOLD signal.

For this paper we aimed to focus on the best analysis method and therefore chose to use a uniform baseline, as the analysis method works independent of a subjects CO_2_/O_2_ baseline. We acknowledge that a this uniform baseline instead of a physiological baseline has limitations regarding the clinical interpretation of the BOLD response, but suits the method usability demonstration aimed here.

Due to the noisy nature of the BOLD signal, DTP and DTB determination needed spatial and temporal smoothing. Similarly to others BOLD fMRI studies, the signal to noise ratio (SNR) in the WM is lower, due to the inhomogeneity of the coil array sensitivity. This low SNR might disturb the response and delays estimations of BOLD signal responses below the noise level. We, therefore, used a spatial 8 × 8 × 8 mm FHWM Gaussian low pass filter and a temporal robust Loess smoothing method with a 32 s sliding – shifted with each TR –window, with which a local regression using weighted linear least squares weights and a 2nd degree polynomial model is performed, assigning lower weight to outliers in the regression. The 16 time points used in this analysis are sufficient to provide a good estimate of the local signal evolution – to determining fitting parameters, and noise – to determine outliers.

The smoothing is performed a posteriori and “centered”, so no additional delay is induced through the smoothing. This smoothing method results in overestimating of the very short DTPs and DTBs. However, it also provides the possibility for robust DTP and DTB calculations.

On the other hand, for the very long DTP and DTB, the accuracy of finding the true physiological DTP and DTB for a calibrated baseline CO_2_ depends mainly on the sufficient length of the CO_2_ increase and return to baseline values (after the stepchange). We have opted for a stepwise CO_2_ change of 80 s since preliminary experiments confirmed such a period would be an adequate duration for most vessels to fully react to the CO_2_ challenge while maintaining high tolerability of subjects to hypercapnia. We extended the CO_2_ baseline to 50 TR (100 s) to allow tissue with potential CO_2_ storage to have a larger DTB than DTP (i.e., to react slower to a decreasing CO_2_ than during an increase). However, in areas of the brain with less vascular density or end‐arteries, for example, periventricular or in the centrum semiovale, DTP durations of around the duration of stepwise increase can result in statistical power artifacts (too short static stepwise plateau, see Figures 2, 3) and potentially result in under‐ or overestimation of the actual maximal CVR for that voxel. Moreover, with 5% CO_2_/95% O_2_ inhalation, (Donahue et al. (2013) found in Moyamoya patients an average of 90 ± 27 s for their TTP measurements, stating that the minimal duration of a CO_2_ pseudo square wave should at least be 2 min Determining the physiological DTP and DTB is limited for voxels approaching DTP values of 80 s and DTB values of 100 s. Comparison to longer CO_2_ pseudo square wave changes will determine the optimal stepwise CO_2_ duration needed to calculate DTP, DTB and CVR_stat_.

## 5 Conclusions

Iterative decomposition and novel determination of different components of the dynamic CO_2_‐BOLD relationship improves sensitivity and reliability of quantitative CVR measurements.

## Conflicts of Interest

JA Fisher is the chief scientist of Thornhill Research Inc., Toronto, Canada (TRI), a spin‐off company from the University Health Network Toronto. The company developed the RespirAct^TM^, which is currently dispensed as a non‐commercial research tool around the world. J. Fierstra has worked as an external consultant at Thornhill Research Inc. from June 2009 till April 2011 while working on his PhD related research projects involving the RespirAct^TM^ system. The other authors report no conflicting interests.
