# Supplementary figures and images for: Iterative analysis of cerebrovascular reactivity dynamic response by temporal decomposition
Source: Brain Behav. 2017 Jul 26;7(9):e00705. doi: 10.1002/brb3.705 (PMC5607533; doi:10.1002/brb3.705)

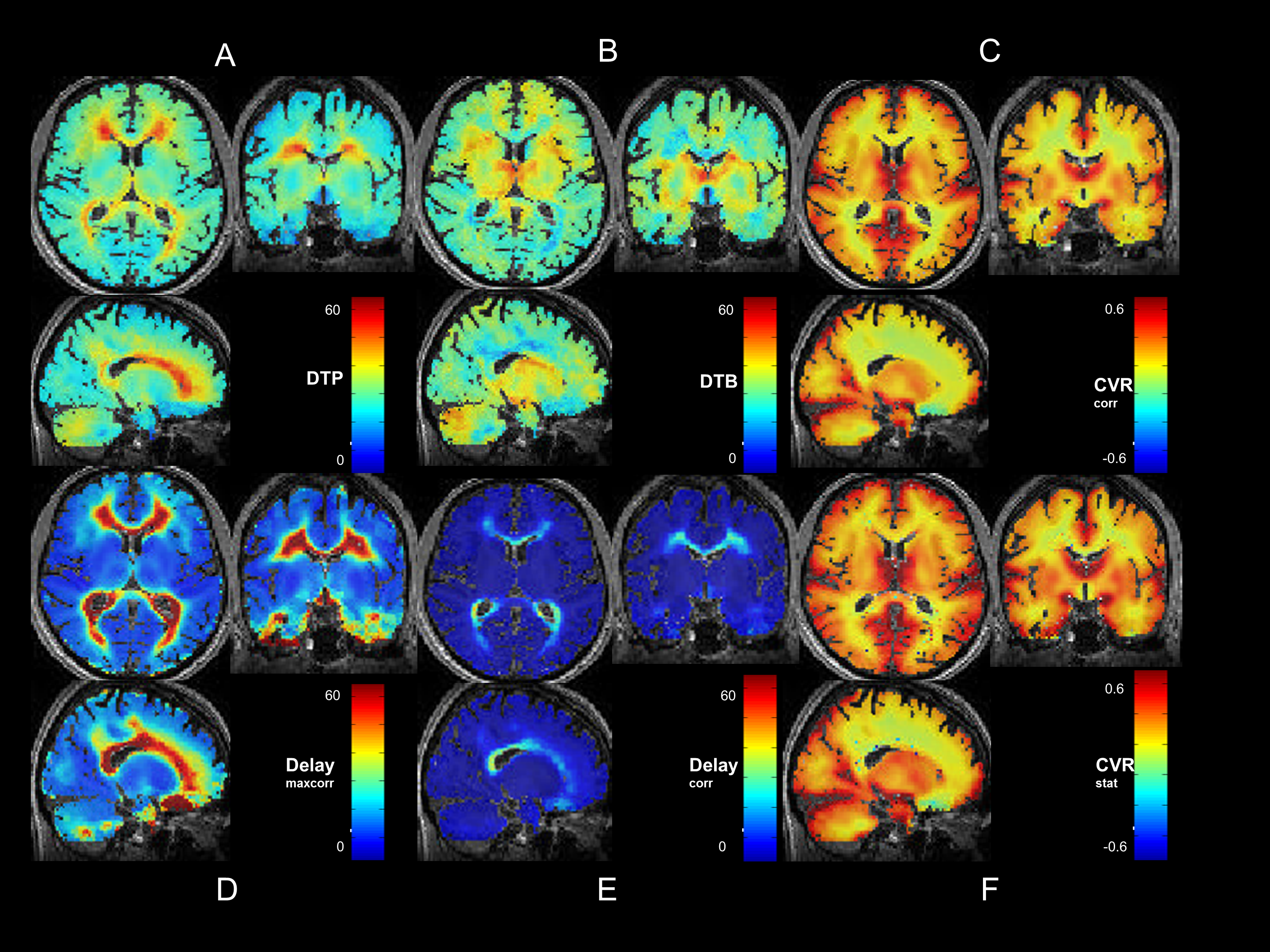

Supplement: Supplementary file 1 [file BRB3-7-e00705-s001.tif]

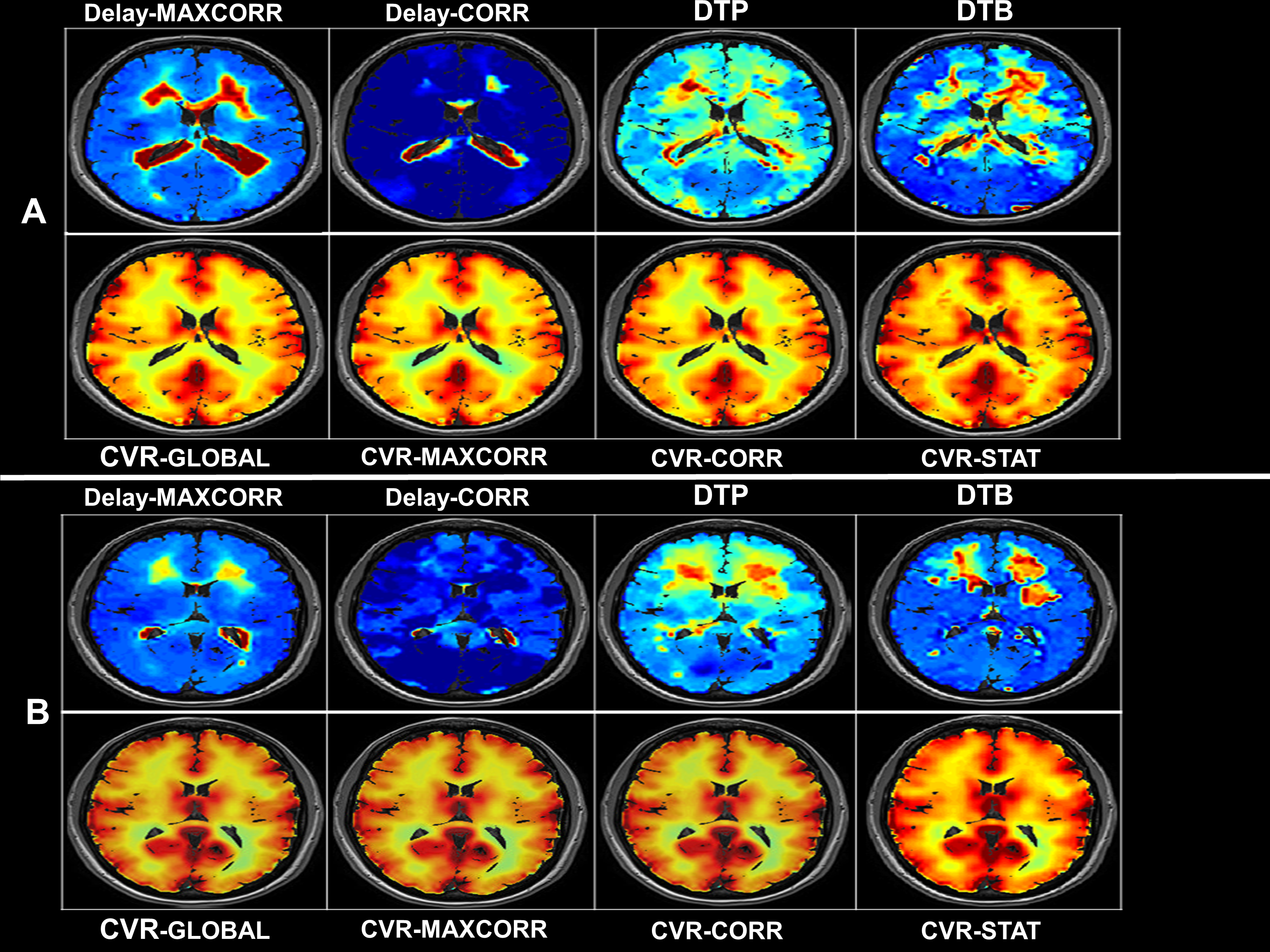

Supplement: Supplementary file 2 [file BRB3-7-e00705-s002.tif]
