# Supplementary material for: Iterative analysis of cerebrovascular reactivity dynamic response by temporal decomposition
Source: Brain Behav. 2017 Jul 26;7(9):e00705. doi: 10.1002/brb3.705 (PMC5607533; doi:10.1002/brb3.705)
Supplement: Supplementary file 3 [file BRB3-7-e00705-s003.docx]

| Table 1: Demographics Reference Atlas | | | |
| --- | --- | --- | --- |
| Age | Subjects (N) | CO2 | Mean ± SD |
| 20 | 12 | O2 baseline  CO2 baseline  O2 step  CO2 step  ΔCO2 | 105±4  40.1±1.3  106±5  48.4±1.6  8.3±1.4 |
| 30 | 8 |  |  |
| 40 | 1 |  |  |
| 50 | 3 |  |  |
| 60 | 1 |  |  |
| Total | 25 |  |  |
